# Environmental concerns and climate change: Need for proactive participation

**DOI:** 10.4103/0019-5278.64606

**Published:** 2010

**Authors:** Ganesh Kulkarni

**Affiliations:** Editor, IJOEM, General Manager, Siemens Ltd., Medicare and OHS, Thane - Belapur Road, Thane - 400 601, Maharashtra, India. E-mail: ganesh.kulkarni@siemens.com

It was 100 years ago that concerns were raised on continued burning of fossil fuels which would contribute to increased levels of carbon dioxide to earth's atmosphere and lead the way for climate change. The rapid industrialization and careless attitude by the mankind lead to environmental deterioration. It is only toward the end of 20^th^ century that Governments and concerned environmental activists started wondering whether inappropriate human actions were influencing the earth's climate.

As we observe World Environment Day on 5 June, we shall put the spotlight on environmental issues and its importance in our role as medical practitioners. With heated discussions during the Copenhagen Summit in 2009 and the recent environmental talks in Bonn, Germany, climate change and other environmental concerns are being recognized by people from diverse fields all over the world. While the climate change skeptics believe that climate change is not an anthropogenic problem, we can see examples, particularly in the developing world of the devastation caused by climate change---cyclones in Orissa and Bangladesh, rising water levels in the Maldives, desertification in north-west Africa, land loss in the small island nations of the Pacific, heat waves in Europe, and changing weather patterns. Global international agreements such as the Kyoto Protocol may have shed some light on the issue of climate change; however, its real impact is being felt in the poorest countries and by the poorest people. In addition to physical challenges, climate change is an ethical challenge because those who are affected most by it are the ones who are least responsible for it. The poor who lack physical, financial, and other resources are most vulnerable to this change and fail to adapt successfully to cope with climate risk. Changing rainfall patterns have rendered farmers helpless and have also left more than a billion people without access to safe drinking water. Natural disasters have created several environmental refugees, particularly in Africa and South Asia.

The negative impacts of climate change on human health are also quite daunting. Evidence has shown that sub-Saharan Africa and coastal areas of the Indian and Pacific ocean will be most seriously affected and the World Health Organization (WHO) estimates that 1,50,000 deaths a year are already being caused by climate change. Climate change is said to affect human health through heat waves and droughts and also through the rapid spread of infectious diseases. It is also expected to affect human health due to deteriorating air quality and malnutrition caused by declining agricultural yield and production. Changes in global temperatures have disproportionately affected the tropics causing deaths directly linked to extreme heat or cold and the depletion of water resources. Rising temperatures have also changed the range of infective parasites, thereby increasing the risk of contracting "vector-borne" diseases like malaria, dengue, yellow fever, and encephalitis. The Intergovernmental Panel on Climate Change (IPCC) has indicated that the global population at a risk of vector-borne malaria due to climate change is slated to increase by 220 million to 400 million in the next century.

The quality of medical care available to the people in the affected regions will therefore play a major role in determining how well these populations are able to adapt to changes in our environment. Clearly, environmental change today poses a huge challenge for the medical community. As practitioners of occupational health services, we must put environmental concerns at the top of our agenda. This can be done by monitoring industrial hygiene, vector control, air quality control, medical waste treatment, hazardous materials management, toxic chemical exposure, and radiological health. Disaster preparedness and timely medical response should also form a key component of our occupation. As medical practitioners it is our duty to press for positive environmental changes within the workplace and even elsewhere. By making medical practice more sensitive to environmental concerns, we have the power to safeguard human health and help populations adapt to changes in the environment.

Governments and businesses have now started adopting a 'green' agenda and promoting environmentally sound practices that reduce their overall carbon footprint. While some may argue that this is enough, the enormity of the problem demands that each one of us do our bit toward conserving the environment. So start today, this very moment and do your bit !

